# Presenting a Case of an Extralobar and Extrapleural Pulmonary Sequestration in a Four-Month-Old Infant

**DOI:** 10.7759/cureus.30331

**Published:** 2022-10-15

**Authors:** Raymond I Okeke, Christian Saliba, Diana Fan, Bernard Parrish, Luke Van Gorp, Caleb Yockey, David Bagley, Shin Miyata, Justin Sobrino, Christopher Blewett

**Affiliations:** 1 General Surgery, Saint Louis University School of Medicine, Saint Louis, USA; 2 Pediatric Surgery, SSM Health Cardinal Glennon Children's Hospital, Saint Louis, USA; 3 Genera Surgery, Saint Louis University School of Medicine, Saint Louis, USA

**Keywords:** congenital lesions, congenital, pulmonary, extrapulmonary sequestration, sequestration, extralobar sequestration

## Abstract

Pulmonary sequestration is a congenital disease formed by embryogenic separation of the lung parenchyma, halting development and function. It has an aberrant blood supply and can provide a nidus for infection and respiratory compromise. It can be diagnosed prenatally with surgical resection after delivery reserved as the best mode of treatment. In literature, six to twelve months is the most optimal time for elective surgical repair giving time for some maturation to withstand single lung ventilation and operation before the risk of infection heightens after 12 months. We present a case of an infant that had an elective repair at four months of age with no postoperative sequelae highlighting that surgeons can perform elective repair sooner than six months of age and that surgical decision-making should be on a case-by-case basis.

## Introduction

Pulmonary sequestration (PS) is a rare congenital disease representing 0.16% of all structural lung diseases and 1.5% of all congenital pulmonary malformations [1]. It comprises a non-functioning mass of pulmonary tissue that becomes separated from the bronchopulmonary tree and gets supplied by one or more aberrant systemic arteries. PS is divided into intralobar pulmonary sequestration (ILPS) and extralobar pulmonary sequestration (ELPS), depending on the presence of complete visceral pleura [1-3]. ILPS shares the same visceral pleura as the originating lung. ELPS has a complete and separate visceral pleura than the neighboring lung and is less common, comprising 15-25% of all pulmonary sequestrations [1,2]. Most ELPS occur between the lower lobe of the left lung and the diaphragm, and its feeding artery mainly originates from the thoracic aorta, abdominal aorta, or other vessels in the systemic circulation [1]. Venous drainage reaches the right atrium via the azygos, hemiazygos vein or vena cava [1]. CT angiography or catheter angiography can assist in identifying the abnormal vessel both for diagnosis and surgical resection [2]. As compared to open thoracotomy, video-assisted thoracoscopic surgery has increasingly become the preferred operating procedure in treating pulmonary sequestration due to less operative pain and faster recovery [3]. We report an even rarer case of pulmonary sequestration which turned out to be extralobar and extrapleural.

## Case presentation

**Figure 1:**
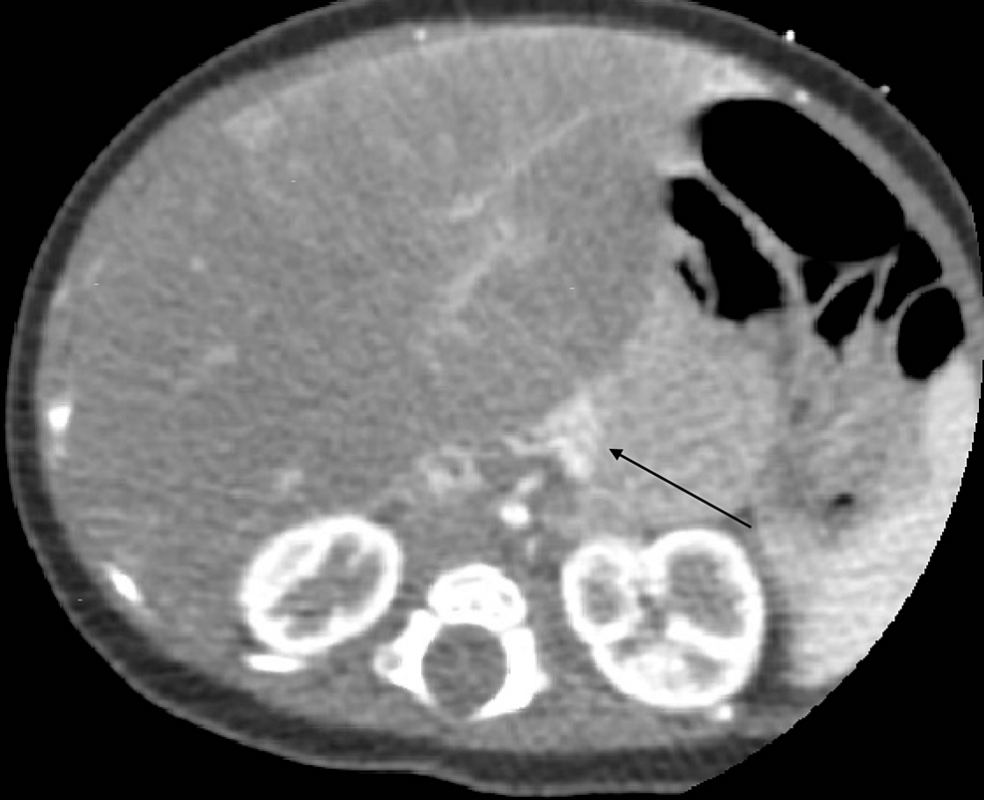
Axial CT view showing extralobar pulmonary sequestration (black arrow)

**Figure 2:**
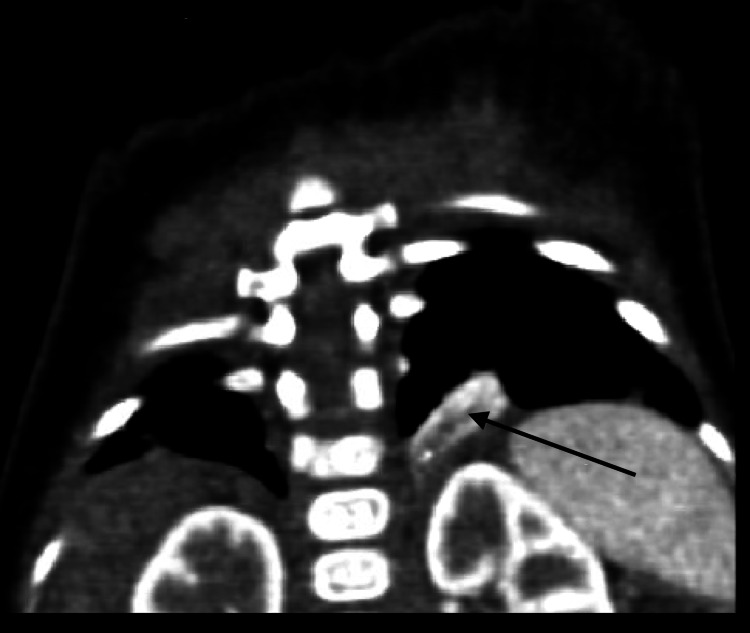
Coronal CT view showing extralobar pulmonary sequestration (black arrow)

The patient is a four-month-old female with a left lower lobe congenital pulmonary airway malformation (CPAM) diagnosed prenatally on ultrasound imaging who underwent interval imaging three months after birth with computed tomography (CT) angiography of the chest, which confirmed a persistent, left lower lobe lesion (Figures 1, 2) with a systemic feeding vessel originating from the aorta. We performed elective surgical resection at four months of age via thoracoscopy inspecting the entire left hemithorax. The left upper and lower lobes, as well as the inferior pulmonary ligament, were normal. However, we noticed a mobile lesion at the base of the thoracic cavity, medially and below a pleural investment. The parietal pleural was incised and dissected from the underlying lesion, which resembled aberrant lung tissue. The mass was in fact outside the parietal pleural making it extrapleural. The systemic feeding vessel was isolated, ligated, and divided using a 5 mm vessel sealer. Next, we applied a 5 mm clip to the stump. The patient tolerated the procedure well. Pathology identified left extrapulmonary sequestration located supradiaphragmatic with a complete visceral pleural making it extralobar. Post-operatively, she had good oxygen saturation levels on room air and had good urine output with one bowel movement after breastfeeding. We removed the left chest JP drain the day after surgery and discharged the patient home.

## Discussion

Pulmonary sequestration (PS) is a rare congenital pulmonary malformation characterized by a non-functioning lung tissue mass that does not communicate with the rest of the tracheobronchial tree [4]. It receives its blood supply from the systemic circulation with or without a separate venous drainage and comprises 0.15 to 6.4% of all congenital lung malformations [2,3]. It was first described in 1861 by Rokitansky via a fraction theory in which there is a separation of normally developing lung tissue from the rest of the lung during development which subsequently becomes non-functional [4-6]. Pryce then described it in 1946 via a traction theory whereby traction on lung tissue by maturing primitive systemic vasculature results in sequestration when an aberrant early interruption of pulmonary arterial development occurs [4,6-7]. With this theory, sequestration can be extralobar if traction occurs early enough in development to cause the growth of visceral pleura around the sequestered lung tissue or intralobar if traction occurs after the lung pleura has already formed [8]. Pulmonary sequestrations occur in the bronchopulmonary malformation spectrum, which includes other malformations like congenital lobar hyperinflation, bronchogenic cyst, congenital pulmonary airway malformation (CPAM), bronchial atresia, and arteriovenous malformation [6,9] based on various aberrations encountered during embryogenic development.

Pulmonary sequestrations occur primarily in the left lower lobe [4]. Intralobar pulmonary sequestration (ILPS) (75-85%) occurs more commonly than extralobar pulmonary sequestration (ELPS) (15-25%) [2,3,10]. ELPS, with its distinct separate visceral pleural covering, is mostly identified in infancy and has a higher incidence in males [1,11]. It can be located at the left posterior costophrenic groove or below the diaphragm [12] and is more likely to occur with gastrointestinal abnormalities, diaphragmatic hernias, and congenital bronchopulmonary foregut malformations [13,14]. Despite our patient having an ELPS, there was no parietal pleural covering, and the lesion had only a visceral pleura; the lesion in our case was extralobar and extrapleural. ILPS occurs primarily at the left posterior basal segment [3]. Arterial blood supply to ELPS is anomalous and originates from systemic circulation. Supply comes mostly from the descending thoracic aorta, the celiac axis or splenic artery, the abdominal aorta, and intercostals [15,16]. Blood supply can rarely come from the subclavian artery, pericardium, coronary arteries, or the following abdominal vessels: left gastric, superior mesenteric, phrenic, and renal arteries [15]. In our patient, arterial supply was from the abdominal aorta. Venous drainage is via systemic circulation; the azygos vein, hemiazygos vein, vena cava, or directly to the right atrium. It could also be into the intercostal, left mammary, innominate, or lower pulmonary veins [3,4,12,17].

In literature, bilateral pulmonary sequestration, co-existing intralobar and extralobar, and pulmonary sequestration connecting with the digestive tract exist in addition to intralobar and extralobar presentations [1,2,8,14,18]. While ILPS are prone to recurrent pneumonia [3], ELPS can also have potential infection risks with the rate varying from 16 to 31% [2]. Additionally, a small number of patients with ELPS can present with necrosis from pedicle torsion [2]. Prenatal diagnosis is by fetal Doppler ultrasound to confirm systemic arterial supply as early as 16 to 18 weeks [3]. Records for our patient show that our patient had ultrasound imaging completed by 32 weeks, but there was no mention of the specific time of ultrasound diagnosis. Computed tomography (CT), CT angiography (CTA) imaging, or magnetic resonance angiography (MRA) scan postnatally diagnose ELPS [3,16]. ELPS appears as a well-defined mass that does not contain air on CT imaging. PS can be confused with lung cysts, lung abscesses, benign lung tumors, and lung cancer and thus has a high rate of misdiagnosis [19].

Surgical resection is recommended for treatment and is best performed at six to twelve months of age if elective [4,16]. Children less than six months have poor intraoperative tolerance of single lung ventilation during resection [16]. In addition, the probability of infection increases over twelve months of age, creating more perioperative complications with operating in this period [4,16]. Our patient was asymptomatic throughout, and able to tolerate the procedure at four months without any postoperative complications. We proceeded with the surgery at this time because the mother advocated for earlier surgery after the prenatal diagnosis despite the absence of symptoms. With ELPS, pedicle torsion and necrosis may require urgent surgical intervention [12]. Video-assisted thoracoscopic surgery is superior to the thoracotomy approach in terms of postoperative outcomes, hospital stay, and postoperative pain [10,19]. Preoperative arterial endovascular embolization [7], uniportal resection [18], and indocyanine green imaging to identify the sequestration [20] are newer adjuncts to ensure precision, safety, and minimal invasion with the best outcomes seen in asymptomatic patients. Surgery timing is selected on a case-by-case basis but with the goal of preventing or treating cases of recurrent pneumonia and lung abscesses.

## Conclusions

We present this rare case of a left extralobar extrapleural pulmonary sequestration which only has visceral pleural covering without a parietal pleural. Despite this atypical presentation, elective management remains the same as an ELPS. Timing for surgery should be on a case-by-case basis.
